# Efficacy and safety of combination therapy with pirfenidone and nintedanib in patients with idiopathic pulmonary fibrosis

**DOI:** 10.3389/fphar.2023.1301923

**Published:** 2023-12-12

**Authors:** Jin-Young Huh, Jae Ha Lee, Jin Woo Song

**Affiliations:** ^1^ Department of Pulmonary and Critical Care Medicine, Asan Medical Center, University of Ulsan College of Medicine, Seoul, Republic of Korea; ^2^ Division of Pulmonary, Allergy and Critical Care Medicine, Department of Internal Medicine, Chung-Ang University Gwangmeyong Hospital, Chung-Ang University College of Medicine, Gwangmyeong, Republic of Korea; ^3^ Division of Pulmonology and Critical Care Medicine, Department of Internal Medicine, Inje University Haeundae Paik Hospital, Inje University College of Medicine, Busan, Republic of Korea

**Keywords:** idiopathic pulmonary fibrosis, antifibrotic agents, pirfenidone, nintedanib, combination therapy

## Abstract

**Background:** Recent studies have suggested that combination therapy with pirfenidone and nintedanib is safe and tolerable in patients with idiopathic pulmonary fibrosis (IPF). However, data from real-world practice are limited. Thus, we aimed to investigate the safety and efficacy of this combination therapy in patients with IPF in a real-world setting.

**Methods:** A multicenter retrospective cohort study was conducted to investigate the safety and efficacy of combination therapy with pirfenidone and nintedanib in 45 patients with IPF. Incidences of adverse events and rates of lung function decline were compared before and after the combination therapy. Propensity score matching was performed to compare the outcomes between the combination and monotherapy groups.

**Results:** The mean age of the patients was 68.8 years, and 82.2% of them were male. The median follow-up duration after combination therapy was 12.1 months. The majority of the patients (97.8%) received nintedanib as an add-on to pirfenidone. The most common adverse events after the combination therapy were diarrhea and anorexia. Pirfenidone or nintedanib was stopped in 12 patients owing to gastrointestinal AEs, lung transplantation, or financial problems. In patients with serial lung function data, the rate of decline in the forced vital capacity was significantly reduced after the combination therapy. In the matched analysis, the combination group had a higher incidence of diarrhea than the monotherapy group without an increase in serious adverse events; however, the two groups had similar changes in forced vital capacity (FVC).

**Conclusion:** The combination of pirfenidone and nintedanib in patients with IPF has the potential to reduce the rate of FVC decline. However, in the matched analysis, FVC decline was comparable between the patients on combination therapy and those on monotherapy. The incidence of certain adverse events, particularly diarrhea, was higher with combination therapy, but serious adverse events were similar between the groups.

## Introduction

Idiopathic pulmonary fibrosis (IPF) is a chronic progressive fibrosing interstitial pneumonia of unknown cause (Raghu et al., 2011). In 2014, disease-modifying antifibrotic agents, pirfenidone and nintedanib, were introduced for IPF treatment. The benefits of antifibrotic therapy include a slower rate of decline in forced vital capacity (FVC) (Noble et al., 2011; King et al., 2014) and reduced mortality (Noble et al., 2016; Yoon et al., 2019). Nevertheless, IPF continues to be characterized by progressive dyspnea and a poor prognosis (Raghu et al., 2018) because the treatment can only delay IPF progression and cannot halt or reverse the damage (Richeldi et al., 2017). Although clinical trials for novel drugs are currently ongoing, no other medications aside from pirfenidone and nintedanib have been approved (Lee et al., 2019). Thus, novel treatment strategies, such as combination treatment, are necessary.

Pirfenidone inhibits the proliferation of fibroblasts and activates the transforming growth factor beta-induced signaling pathway (Conte et al., 2014); however, its exact mechanisms of action remain unclear. Nintedanib is a tyrosine kinase inhibitor that targets multiple growth factor receptors associated with fibrogenesis (Hilberg et al., 2008). The different mechanisms of action of these two drugs in the fibrotic cascade suggest the potential for additive or synergistic effects. Two prospective studies reported that combination therapy with pirfenidone and nintedanib is safe and tolerable (Flaherty et al., 2018; Vancheri et al., 2018). Moreover, combination therapy further slowed the rate of decline in FVC (Vancheri et al., 2018). However, real-world studies assessing its efficacy are scarce (Hisata et al., 2021).

Real-world studies, such as the present investigation, offer advantages in terms of generalizability to a broader patient population, reflecting the complexity and heterogeneity of clinical practice. However, they are more susceptible to confounding variables due to the lack of randomization and strict inclusion criteria characteristic of prospective cohort studies. Standardization of the intervention and data collection is more difficult in clinical practice settings, which may affect the reliability and validity of the results (Beaulieu-Jones et al., 2020). To address these limitations, our study used propensity score matching to compare the combination therapy and monotherapy groups, as well as a before-and-after analysis of the combination therapy. In this study, we aimed to provide further evidence on the safety and efficacy of combination therapy with pirfenidone and nintedanib in patients with IPF using real-world data.

## Materials and methods

### Study design and population

From January 2004 to February 2019, 2,388 patients were diagnosed with IPF at Asan Medical Center, Seoul, Republic of Korea, and from January 2018 to December 2020, 158 patients were diagnosed at Haeundae Paik Hospital. Among the patients who underwent antifibrotic treatment during follow-up (*n* = 1,101), 1,056 who received a single antifibrotic agent were excluded; 45 patients who received combination therapy with pirfenidone and nintedanib were finally included in this study (Supplementary Figure S1). All patients were diagnosed with IPF in accordance with the diagnostic criteria of the American Thoracic Society (ATS)/European Respiratory Society (ERS)/Japanese Respiratory Society/Latin American Thoracic Association (Raghu et al., 2018). The included cases were also matched with the existing IPF cohort of 1,360 patients diagnosed from 2004 to 2017 at Asan Medical Center to compare the clinical outcomes between combination and monotherapy patients (Kang et al., 2020). The 45 cases included in this study were propensity score matched with 64 patients who received pirfenidone alone. The matching variables were age, sex, FVC, and diffusing capacity for carbon monoxide (DL_CO_). This study protocol was approved by the Institutional Review Board of Asan Medical Center (IRB No. 2021-0183) and Haeundae Paik Hospital (IRB No. 2021-05019). Informed consent was waived owing to the retrospective design of the study.

### Data collection

The clinical and survival data of all patients were obtained from medical records and/or National Health Insurance Service of Korea records. Patients were followed from the index date until death or censoring (7 July 2022). The results of patient pulmonary function tests (PFT) and 6-min walk tests (6MWT) performed 6 months before and after initiating combination therapy, as well as their adverse event (AE) profiles during the antifibrotic treatment, were obtained. In the matched analysis, the index date for the monotherapy group was the day an antifibrotic was started, whereas that for the combination therapy group was the day the second antifibrotic was added. Spirometric parameters (Miller et al., 2005), DL_CO_ (Macintyre et al., 2005; Graham et al., 2017), and total lung capacity (Wanger et al., 2005) were measured in accordance with the ERS/ATS recommendations, and the results are presented as percentages of the normal predicted value. The 6MWT was performed following previously published guidelines (Singh et al., 2014).

### Safety evaluation

To assess AEs related to combination therapy, we compared the occurrence of AEs before and after initiating combination therapy. AEs that presented before starting combination therapy and were aggravated afterward were categorized as “aggravation of pre-existing AEs.” AEs that occurred after introducing a new antifibrotic agent were classified as “newly developed AEs.” The number of AEs 6 months after adding an antifibrotic agent was also compared between the combination and monotherapy groups. The AE terms were classified in accordance with the Medical Dictionary for Regulatory Affairs, version 24.0. Serious AEs were defined as any event that resulted in 1) death, 2) life-threatening hospitalization, 3) disability or permanent damage, 4) an intervention to prevent permanent impairment or damage, or 5) other serious medical events. Acute exacerbations were defined in accordance with the criteria suggested by Collard et al. They defined an acute exacerbation as an acute, clinically significant respiratory deterioration within 1 month, characterized by evidence of new widespread alveolar abnormality, that is not fully explained by cardiac failure or volume overload (Collard et al., 2016).

### Efficacy evaluation

Study outcomes included the rate of lung function decline, changes in lung function, and rate of disease progression. The rates of lung function decline were determined by estimating the slope (mL/month) from the linear mixed-effects model. In this model, age, sex, smoking status, BMI, and time were the fixed effects, whereas each person was the random effects. To be included in the analysis of FVC decline, patients had to have repeated measures (at least two) of their FVC both before and after the start of the combination therapy. This criterion was used to ensure that there were enough data points to reliably estimate the rate of decline in lung function. Of the initial 45 patients on combination therapy, 32 met this inclusion criterion. Changes in lung function or 6MWD were defined based on the absolute differences between the baseline and 6-month values [FVC, mL (or 6MWD, m), after 6 months—FVC, mL (or 6MWD, m) at baseline]. The rate of disease progression was defined as ≥5% (DP5) or ≥10% (DP10) absolute decline in FVC % predicted over 6 months. The rates of lung function decline, changes in lung function, and rates of disease progression before and after the administration of combination therapy were compared. In addition, matched patients in the two groups were compared for changes in lung function and rates of disease progression.

### Statistical analysis

Continuous variables are presented as the mean ± standard deviation or as the median and interquartile range (IQR). Categorical variables are stated as numbers (in percent). Student’s *t*-test or Wilcoxon’s rank-sum test was used to compare continuous variables, and *χ* ^2^ or Fisher’s exact test was used to compare categorical variables. Paired *t*-tests were applied to evaluate changes in lung function before and after the combination therapy. Statistical significance was considered at *p* < 0.05 (two-tailed). All statistical analyses were performed using R (version 4.03; R Foundation for Statistical Computing, Vienna, Austria).

## Results

### Baseline characteristics

The median follow-up of the 45 patients after starting the combination therapy was 12.1 months (IQR: 6.8–27.7 months). The mean age was 68.8 years, and 82.2% were male (Table 1). Most of the patients (*n* = 44, 97.8%) were on pirfenidone [mean daily dose: 1627.3 ± 403.7 mg, median duration: 24.7 months (IQR: 15.0–36.0 months)] before receiving add-on nintedanib, except for one patient (daily dose of nintedanib: 200 mg, duration: 4.2 months) (Table 1).

**TABLE 1:** Baseline characteristics of the study population at the initiation of combination therapy.

Characteristics	Total
Number of patients	45
Age, years	68.8 ± 6.2
Male	37 (82.2)
Ever smoker	31 (68.9)
Body mass index, kg/m^2^	24.6 ± 3.4
Time since diagnosis, months	31.0 (17.3–46.3)
Pulmonary function test	
FVC, % predicted	59.6 ± 12.9
DL_CO_, % predicted	43.5 ± 12.6
TLC, % predicted	59.9 ± 9.3
6 MWT	
Distance, m	458.0 ± 115.9
Lowest SaO_2_, %	86.0 ± 4.1
Previous treatment	
Pirfenidone	44 (97.8)
Dose before combination, mg/day	1627.3 ± 403.7
Nintedanib	1 (2.2)
Dose before combination, mg/day (one patient)	200
Median duration of single antifibrotic therapy, months	24.4 (24.5–35.6)
Add-on nintedanib dose, mg/day	229.5 ± 49.8
Add-on pirfenidone dose, mg/day	1800

Data are presented as the mean ± standard deviation, number (%) of patients or median (interquantile range). FVC, forced vital capacity; DLco, diffusing capacity of the lungs for carbon monoxide; TLC, total lung capacity; 6 MWT, 6-min walk test; SaO_2,_ arterial oxygen saturation.

### Adverse events

Most patients (32/45, 71.1%) experienced AEs after receiving combination therapy. The most common AE was diarrhea (18/45, 40.0%), followed by anorexia (17/45, 37.8%) and dyspepsia/abdominal pain (5/45, 11.1%). All events of diarrhea were associated with add-on nintedanib. Most cases of anorexia developed before the combination therapy and persisted or were aggravated after adding another antifibrotic agent (Table 2). Serious AEs occurred in 12 patients (31.1%). The most common serious AE was an acute exacerbation (*n* = 6, 13.3%), followed by focal pneumonia (*n* = 3, 6.7%). Six deaths (13.3%) were caused by acute exacerbation of IPF (3/6, 50.0%), unknown cause (2/6, 33.3%), or septic shock (1/6, 16.7%) (Supplementary Table S1).

**TABLE 2:** Adverse events after combination therapy.

Characteristics	Total AE after combination	Aggravation of pre-existing AE	Newly developed AE
Number of patients	32	14	27
Anorexia	17 (53.1)	13 (86.7)	4 (14.8)
Nausea/vomiting	8 (25.0)	3 (20.0)	5 (18.5)
Dyspepsia/abdominal pain	5 (15.6)	2 (13.3)	3 (16.7)
Diarrhea	18 (56.3)	0 (0.0)	18 (66.7)
Hepatotoxicity >3 × ULN	0 (0.0)	0 (0.0)	0 (0.0)
Constipation	2 (6.3)	0 (0.0)	2 (7.4)
Photosensitivity	1 (3.1)	0 (0.0)	1 (2.7)
Pruritus	2 (6.3)	0 (0.0)	2 (7.4)
General weakness	3 (9.4)	1 (6.7)	2 (7.4)
Insomnia	2 (6.3)	2 (13.3)	0 (0.0)

Data are presented as number (%) of patients. AE, adverse events; ULN, upper limit of normal. Nine patients had both aggravation and new adverse events after initiation of the combination therapy.

Among the 45 patients, 12 (26.7%) withdrew from the combination therapy after a median period of 3.6 months (IQR: 2.7–4.4 months). Nintedanib was discontinued permanently in nine patients owing to financial reasons (*n* = 3), anorexia and diarrhea (*n* = 2), anorexia and dyspepsia (*n* = 1), nausea (*n* = 1), generalized weakness (*n* = 1), or acute exacerbation (*n* = 1). Pirfenidone was stopped in two patients owing to anorexia (*n* = 1) or dyspepsia (*n* = 1). In one patient, both nintedanib and pirfenidone were stopped after lung transplantation.

### Rate of decline in lung function

**FIGURE 1:**
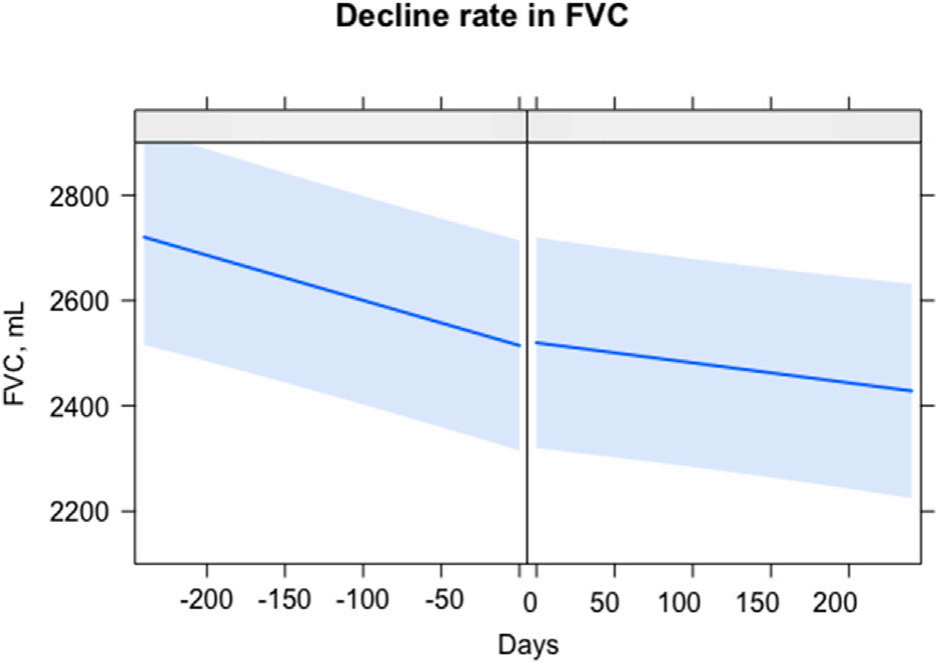
Comparison of lung function decline rate before and after combination therapy. The X-axis is days, with 0 as the start of the combination therapy. The Y-axis is the FVC, mL. The straight blue line represents the fitted values of the FVC. The light blue shading shows the confidence interval. *Abbreviations*: FVC, forced vital capacity.

In 32 patients with serial lung function data (≥2) before and after the start of the combination therapy, the rate of lung function decline was compared 6 months before and after the initiation of the combination therapy (mean total number of PFTs: 6.6 ± 0.8 times). The rate of decline in FVC significantly decreased [−17.7 (before) vs. −10.6 (after) mL/month, *p* = 0.049] after starting the combination therapy (Figure 1). However, the rate of decline in DL_CO_ was not different before and after the start of the combination therapy [−0.005 (before) vs. −0.006 (after) mL CO/min/mm Hg/day, *p* = 0.614].

### Changes in lung function and 6-min walk distance

**FIGURE 2:**
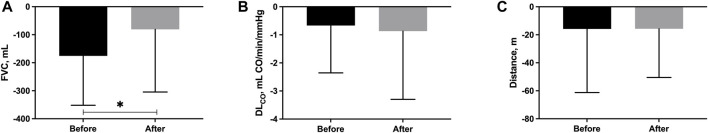
Changes in clinical outcomes before and after the start of combination therapy. **(A)** Changes in the FVC, mL; **(B)** changes in DL_CO_, mL CO/min/mmHg; **(C)** changes in 6 MWD m; Asterisk represents the difference with marginal statistical significance (*p* = 0.074). *Abbreviations*: FVC, forced vital capacity; DL_CO_, diffusing capacity of the lungs for carbon monoxide; 6 MWD, 6-min walking distance.

Changes in FVC, DL_CO_, and 6MWD 6 months before and after the start of the combination therapy were compared. The decline in FVC was reduced after starting the combination therapy [−175.7 (before) vs. −81.0 (after) mL, *p* = 0.074]; however, this result was not statistically significant (Figure 2). Similarly, DL_CO_ [−0.7 (before) vs. −0.9 (after) mL CO/min/mm Hg, *p* = 0.789] and 6MWD [−15.8 (before) vs. −15.7 (after) m, *p* = 0.992] showed no significant changes after starting combination therapy.

**FIGURE 3:**
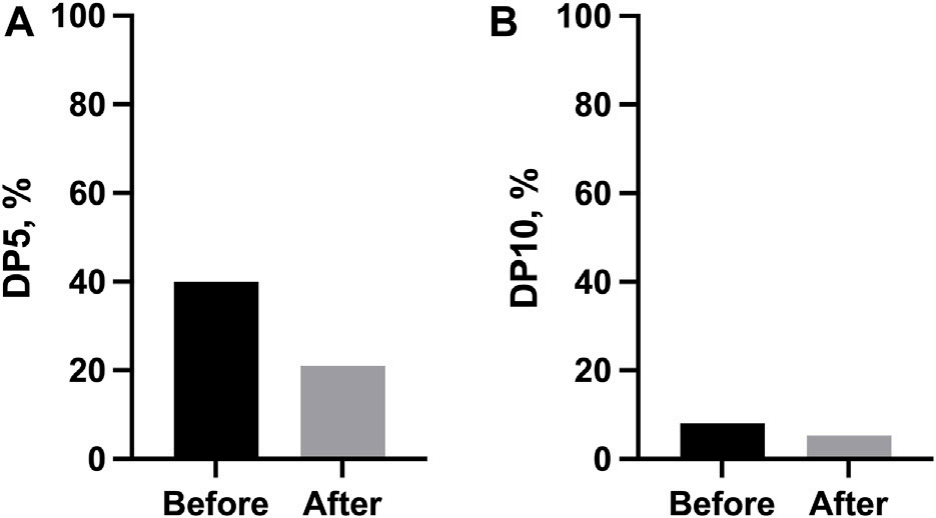
Categorical changes in lung function before and after the start of combination therapy. **(A)** Proportion of disease progression, defined as a 5% absolute decrease in FVC % predicted over 6 months; **(B)** Proportion of disease progression, defined as a 10% absolute decrease in FVC % predicted over 6 months. *Abbreviations*: DP5, disease progression, defined as a 5% absolute decrease in FVC % predicted over 6 months; DP10, disease progression, defined as a 10% absolute decrease in FVC % predicted over 6 months.

The changes in FVC were also categorically assessed as DP5 and DP10 over 6 months. Both DP5 [40.0% (before) vs. 26.3% (after), *p* > 0.999] and DP10 [8.0% (before) vs. 5.3% (after), *p* > 0.087] decreased after the combination therapy, but this change was not statistically significant (Figure 3).

### Combination vs. monotherapy

Of the 45 patients, 32 were matched with 64 patients in a previously existing IPF cohort who continued with monotherapy. The cohort included 1,360 patients; their mean age was 66.4 years, and 81.3% were male [median follow-up duration: 27.3 months (IQR: 12.5–55.3)] (Supplementary Table S2). After matching, the mean ages of the patients from the monotherapy and combination therapy groups were 68.4 and 68.5 years, respectively (*p* = 0.942). The percentage of males was 93.8% in both groups, and the BMIs, FVCs, and DL_CO_ values of the patients were comparable (Supplementary Table S3).

In the matched analysis, the median follow-up periods for the combination and monotherapy groups were 9.1 and 17.8 months, respectively (*p* < 0.001). At 6 months after treatment, the combination group experienced more frequent diarrhea (43.8% vs. 14.1%; *p* = 0.001) than the monotherapy group. In contrast, the incidence of pruritus was higher in the monotherapy group (31.2% vs. 6.2%, *p* = 0.013) than in the combination group (Table 3). With regard to treatment efficacy, no significant differences in the changes in FVC [−45.7 (combination therapy) vs. −44.8 (monotherapy) mL/6 months*; p* = 0.715], DL_CO_ (−1.0 vs. −0.4 mL CO/min/mm Hg/6 months; *p* = 0.093) or 6 MWD (−0.9 vs. −6.5 m/6 months; *p* = 0.897) were observed between the two groups (Supplementary Figure S2). Moreover, DP5 [21.4% (combination therapy) vs. 26.8% (monotherapy), *p* = 0.789] and DP10 (7.1% vs. 7.1%, *p* > 0.999) were comparable between the two groups.

**TABLE 3:** Comparison of adverse events between the combination therapy and matched monotherapy groups.

Adverse events	Combination therapy	Monotherapy	*p*-value
Number of patients	32	64	
Patients with adverse events	24 (75.0)	53 (82.8)	0.526
Diarrhea	14 (43.8)	9 (14.1)	0.003
Anorexia	10 (31.2)	34 (53.1)	0.070
Nausea/vomiting	4 (12.5)	8 (12.5)	>0.999
Dyspepsia/abdominal pain	2 (6.2)	2 (3.1)	0.857
Constipation	2 (6.2)	1 (1.6)	0.534
Pruritus	2 (6.2)	20 (31.2)	0.013
Insomnia	1 (3.1)	0 (0.0)	0.722
Photosensitivity	1 (3.1)	9 (14.1)	0.194
General weakness	1 (3.1)	13 (20.3)	0.052
Rash	0 (0.0)	1 (1.6)	>0.999
Dizziness	0 (0.0)	0 (0.0)	>0.999
Serious adverse events	3 (9.4)	2 (3.1)	0.417
Acute exacerbation	2 (6.2)	0 (0.0)	
Death	1 (3.1)	0 (0.0)	
Pneumonia	0 (0.0)	2 (3.1)	

Data are presented as the number (%) of patients.

## Discussion

Our study showed that in patients with IPF taking an antifibrotic agent at a stable dose, the add-on of another antifibrotic agent decreased the rate of decline in FVC. Most patients (71.1%) reported AEs, and 26.7% stopped taking one of the antifibrotic agents during the follow-up. In the matched cohorts, compared to the monotherapy, the combination therapy did not induce more AEs except for diarrhea. Our study contributes to the limited real-world evidence on the concomitant use of pirfenidone and nintedanib in the treatment of IPF. We have improved the rigor of our analysis by comparing safety and efficacy, not only before and after the initiation of combination therapy, but also by using propensity score matching with the monotherapy cohort to reduce bias.

In our study, the most common AEs were aggravation of anorexia and the development of diarrhea. The discontinuation rate was 26.7%, and the most common causes were gastrointestinal AEs. Our findings are comparable to those of previous studies (Flaherty et al., 2018; Vancheri et al., 2018). Flaherty et al. (Flaherty et al., 2018) conducted a prospective single-arm study involving 89 patients with IPF treated with a stable pirfenidone dose (1,602–2,403 mg/day for ≥16 weeks) and observed that 98.9% of patients who added nintedanib to their ongoing pirfenidone treatment experienced AEs, the most common of which was diarrhea (42.7%). During the 24-week period, 26% had interrupted treatment, and 15% discontinued the treatment permanently owing to AEs (Flaherty et al., 2018). Vancheri et al. (Vancheri et al., 2018) performed a randomized trial involving 105 patients with IPF who were divided into a nintedanib plus pirfenidone group and a nintedanib-alone group. All participants received nintedanib 150 mg twice a day for a run-in period of 4-5 weeks. AEs were observed in 88.7% and 88.2% of the patients in the combination and nintedanib-alone groups, respectively, during the 12-week trial period. Diarrhea was frequently observed in both groups [37.7% (combination) vs. 31.4% (nintedanib alone)]. In addition, 35.3% of the patients in the combination group did not complete the planned treatment, discontinuing one or both of the antifibrotics. Unexpected AEs associated with the administration of both drugs were not observed, suggesting that the combination therapy has acceptable safety and tolerability.

Three patients in our study discontinued nintedanib for financial reasons, a decision influenced by the reimbursement policies in South Korea. The national health insurance system covers the entire population (Sohn and Jung, 2016) and provides reimbursement for pirfenidone for patients with IPF. In contrast, nintedanib is not reimbursed, resulting in out-of-pocket costs for patients (approximately $2,300 per month), which limits access to the drug. As a result, three patients in our cohort were unable to continue their treatment with nintedanib due to these financial constraints.

In this study, lung function was better maintained after adding an additional antifibrotic agent. The declines in FVC were also smaller after starting combination therapy. Previous studies support our findings (Flaherty et al., 2018; Vancheri et al., 2018). Flaherty et al. (Flaherty et al., 2018) reported that the decline in FVC was smaller during combined treatment with pirfenidone and nintedanib than the historical value closest to 6 months prior to screening [rate of decline: 0.8 (historical) vs. 0.4 (after combination therapy) % predicted per 24 weeks]. Vancheri et al. (Vancheri et al., 2018) reported that the combination therapy group showed a smaller decline in FVC than the nintedanib-alone group (3.6 vs. −48.0 mL per 12 weeks). The current lines of evidence consistently suggest additional benefits of the combination therapy in decelerating IPF progression in terms of FVC, at least in the short term.

Here, we matched the combination therapy group with a monotherapy group for comparison. Over 6 months, no significant changes in physiologic parameters were observed. Among all of the adverse events observed, diarrhea was the only one that occurred more frequently in the combination therapy group than in the monotherapy group. Diarrhea has been associated with nintedanib more frequently than pirfenidone (Richeldi et al., 2014; Crestani et al., 2019). However, serious AEs caused by diarrhea were not observed in the present study. A previous study involving add-on pirfenidone reported a lesser decline in FVC after combination therapy compared with monotherapy (Vancheri et al., 2018). However, in the present study, we did not observe significant differences in FVC between the two groups, even after propensity score matching. The lack of differences could be due to a longer time from IPF diagnosis to the index date in the combination therapy group [859.0 (combination therapy) vs. 135.5 (monotherapy) days, *p* < 0.005]. In addition, selection bias may be present in the combination therapy group, considering that the patients in this group may have been prescribed an additional antifibrotic drug owing to a poor response to the initial treatment.

This study has some limitations. First, it is a retrospective observational study conducted in a Korean population only. The unavoidable selection bias may hinder the generalizability of our findings. However, the baseline characteristics of our cohort were similar to those of cohorts in other studies (Flaherty et al., 2018; Vancheri et al., 2018). Second, most patients included in this study were on pirfenidone before the addition of nintedanib. The AE profiles may be distinct in patients who received pirfenidone as an add-on to nintedanib. Nonetheless, two previous prospective studies that differed in the order of antifibrotic treatment showed similar AE profiles after their combination (Flaherty et al., 2018; Vancheri et al., 2018). Third, the number of included patients was small, leading to statistically insignificant results. However, we found significant differences in the decline rate in FVC before and after the initiation of the combination therapy.

## Conclusion

In conclusion, our study suggested that in patients with IPF receiving stable doses of an antifibrotic agent, administering another antifibrotic agent has the potential to decrease the rate of lung function decline with acceptable safety profiles. Despite an increase in the frequency of diarrhea with the combination therapy, its SAE profiles were similar to those seen with monotherapy. Thus, combination therapy with pirfenidone and nintedanib may be a feasible option to decelerate IPF progression.

## Data Availability

The original contributions presented in the study are included in the article/Supplementary Material, further inquiries can be directed to the corresponding author.
