# Supplementary material for: Efficacy and safety of combination therapy with pirfenidone and nintedanib in patients with idiopathic pulmonary fibrosis
Source: Front Pharmacol. 2023 Dec 12;14:1301923. doi: 10.3389/fphar.2023.1301923 (PMC10773730; doi:10.3389/fphar.2023.1301923)
Supplement: Supplementary file 1 [file DataSheet1.docx]

**SUPPLEMETARY MATERIAL**

**Efficacy and safety of combined therapy with pirfenidone and nintedanib in patients with idiopathic pulmonary fibrosis**

Jin-Young Huh, Jae Ha Lee, Jin Woo Song

Supplementary Table 1. Serious adverse events that occurred after the start of combination therapy

| Characteristics | Total |
| --- | --- |
| Total patients | 45 |
| Patients who experienced SAEs | 14 (31.1) |
| Acute exacerbation | 3 (6.7) |
| Pneumonia | 3 (6.7) |
| Pneumomediastinum | 1 (2.2) |
| Death | 6 (13.3) |
| Causes of death |  |
| Acute exacerbation | 3 (6.7) |
| Unknown | 2 (4.5) |
| Septic shock | 1 (2.2) |

Data are presented as the number (%) of patients. SAE, serious adverse events

Supplementary Table 2. Characteristics of the IPF cohort included in the matched analysis

| Characteristics | Total |
| --- | --- |
| Number of patients | 1,360 |
| Age, years | 66.4 ± 8.0 |
| Sex, male | 1105 (81.3) |
| Body mass index, kg/m^2^ | 24.3 ± 3.1 |
| Pulmonary function test |  |
| FVC, % of predicted | 69.9 ± 16.1 |
| TLC, % of predicted | 70.5 ± 14.8 |
| DL_CO_, % of predicted | 56.7 ± 17.8 |
| Follow-up duration, months | 27.3 (12.5–55.3) |

Data are presented as the mean ± standard deviation or number (%) of patients or median (interquartile range).

*Abbreviations*: IPF, idiopathic pulmonary fibrosis; FVC, forced vital capacity; TLC, total lung capacity; DL_CO_, diffusing capacity of the lungs for carbon monoxide

Supplementary Table 3. Baseline characteristics of patients from the combination therapy and matched monotherapy groups

| Adverse events | Combination Therapy | Monotherapy | *p* value |
| --- | --- | --- | --- |
| Number of patients | 32 | 64 |  |
| Age | 68.5 ± 6.0 | 68.2 ± 5.9 | 0.866 |
| Male | 30 (93.8) | 60 (93.8) | >0.999 |
| Body mass index, kg/m^2^ | 25.0 ± 3.2 | 24.7 ± 2.4 | 0.710 |
| Pulmonary function test |  |  |  |
| FVC, % predicted | 60.8 ± 11.3 | 60.3 ± 11.4 | 0.848 |
| DL_CO_, % predicted | 47.5 ± 10.1 | 44.4 ± 10.5 | 0.179 |
| Median time from IPF diagnosis, days | 859.0 (460.5–1432.0) | 135.5 (21.0–768.5) | <0.005 |

Data are presented as the mean ± standard deviation or number (%) of patients.

*Abbreviations*: FVC, forced vital capacity; DL_CO_, diffusing capacity of the lungs for carbon monoxide

Supplementary Figure 1. Flow chart of patients included in the study





*Abbreviations*: IPF, idiopathic pulmonary fibrosis; AMC, Asan Medical Center

Supplementary Figure 2. Comparison of clinical outcomes between patients from the combination therapy and matched monotherapy groups


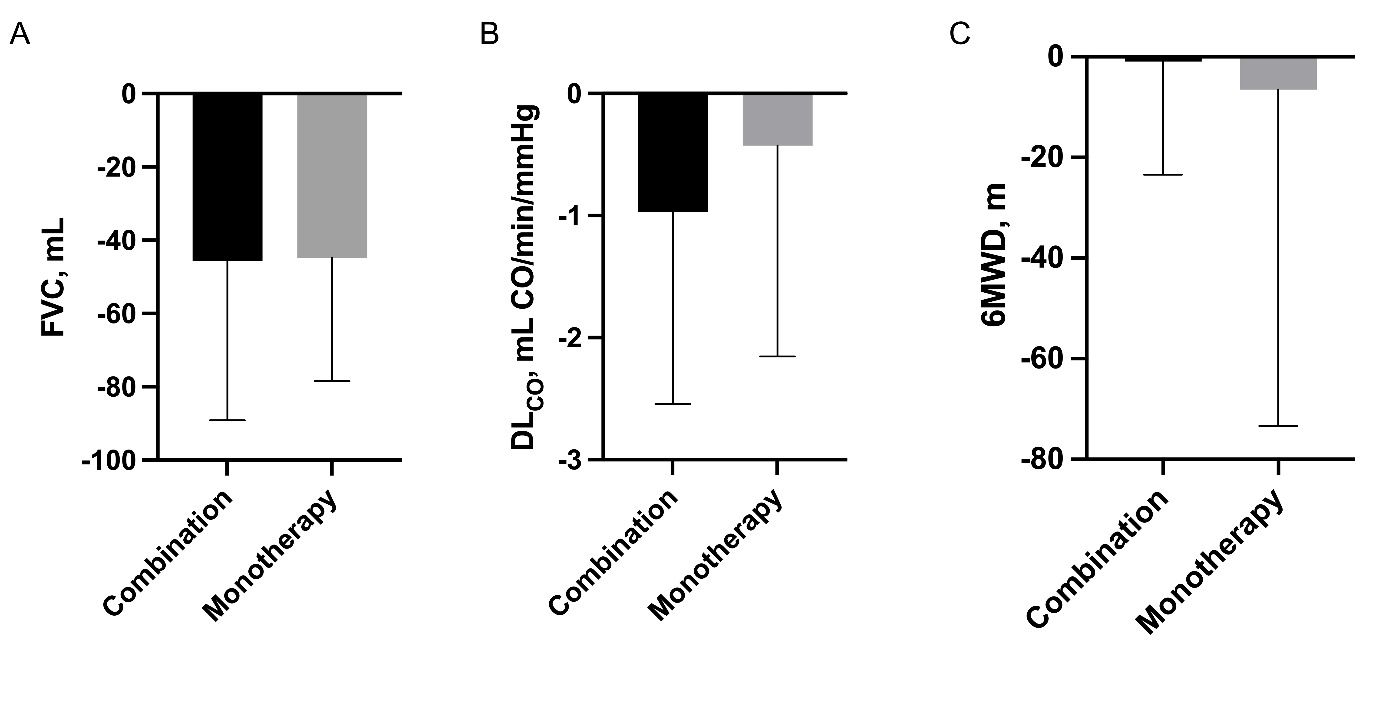


(A) Changes in FVC, mL; (B) changes in DL_CO_, mL CO/min/mmHg; (C) changes in 6MWD, m

*Abbreviations*: FVC, forced vital capacity; DL_CO_, diffusing capacity of the lungs for carbon monoxide; 6MWD, 6-min walking distance
